# Supplementary material for: Causal interplay between lactose intolerance and gut microbiota: a combined bidirectional Mendelian randomization and in vivo validation study
Source: Front Nutr. 2026 Jun 1;13:1803337. doi: 10.3389/fnut.2026.1803337 (PMC13265576; doi:10.3389/fnut.2026.1803337)
Supplement: Supplementary file 1 [file Data_Sheet_1.zip › supplementary materials/Forward/leave one out cross validation/ebi-a-GCST90027524.finngen_R12_E4_LACTONAS.pdf]

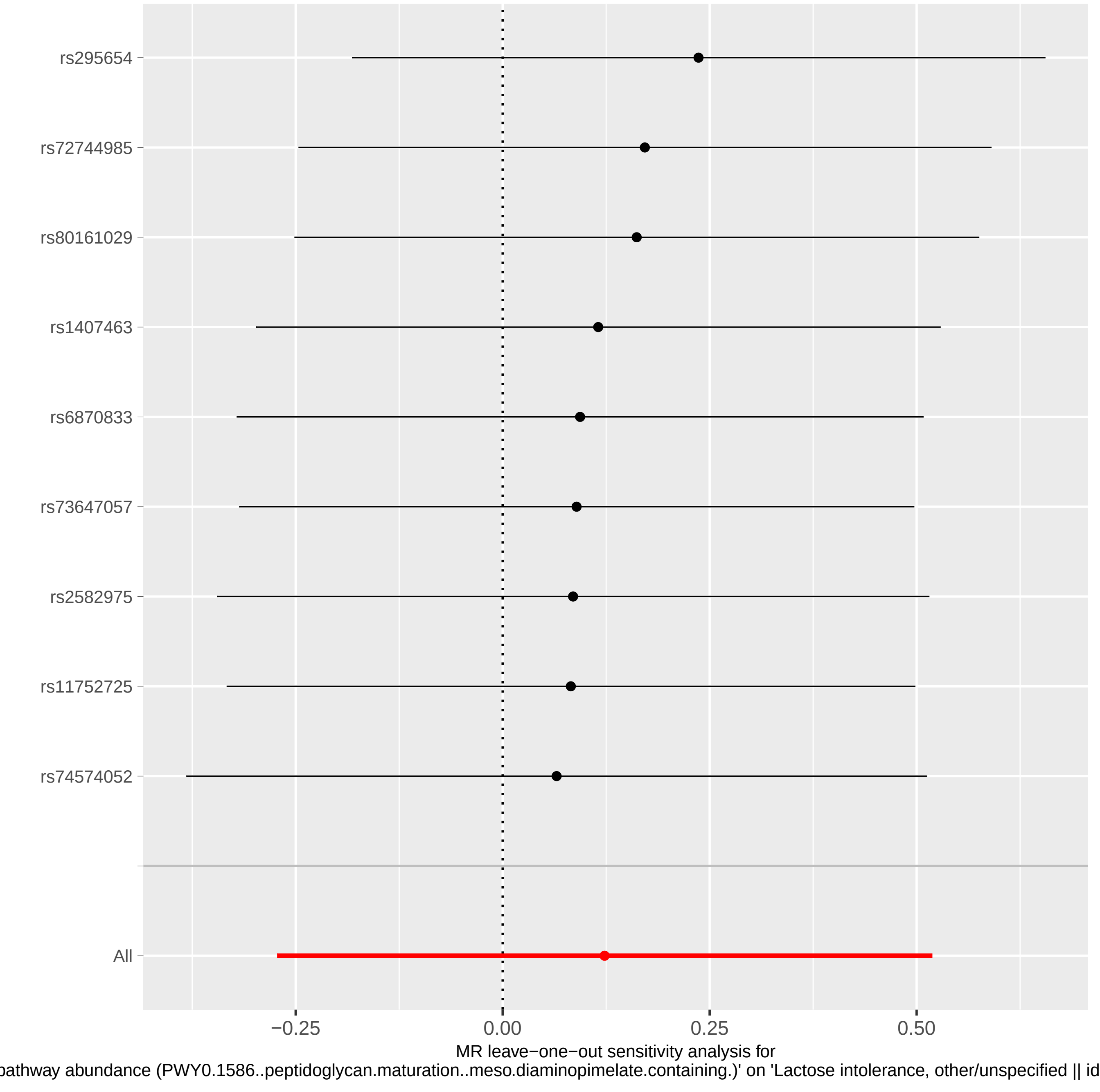

rs295654
rs72744985
rs80161029
rs1407463
rs6870833
rs73647057
rs2582975
rs11752725
rs74574052
All
−0.25
0.00
0.25
0.50
MR leave−one−out sensitivity analysis for
pathway abundance (PWY0.1586..peptidoglycan.maturation..meso.diaminopimelate.containing.)' on 'Lactose intolerance, other/unspecified || id
